# Tertiary Lymphoid Structures in the Central Nervous System: Implications for Glioblastoma

**DOI:** 10.3389/fimmu.2021.724739

**Published:** 2021-09-01

**Authors:** Tiarne van de Walle, Alessandra Vaccaro, Mohanraj Ramachandran, Ilkka Pietilä, Magnus Essand, Anna Dimberg

**Affiliations:** Department of Immunology, Genetics and Pathology, Science for Life Laboratory, The Rudbeck Laboratory, Uppsala University, Uppsala, Sweden

**Keywords:** tertiary lymphoid structure, immunotherapy, glioma, central nervous system, brain, glioblastoma

## Abstract

Glioblastoma is the most common and aggressive brain tumor, which is uniformly lethal due to its extreme invasiveness and the absence of curative therapies. Immune checkpoint inhibitors have not yet proven efficacious for glioblastoma patients, due in part to the low prevalence of tumor-reactive T cells within the tumor microenvironment. The priming of tumor antigen-directed T cells in the cervical lymph nodes is complicated by the shortage of dendritic cells and lack of appropriate lymphatic vessels within the brain parenchyma. However, recent data suggest that naive T cells may also be primed within brain tumor-associated tertiary lymphoid structures. Here, we review the current understanding of the formation of these structures within the central nervous system, and hypothesize that promotion of tertiary lymphoid structures could enhance priming of tumor antigen-targeted T cells and sensitize glioblastomas to cancer immunotherapy.

## Introduction

Glioblastoma (GBM) is a grade IV astrocytoma with dismal prognosis. The overall survival is only 12-15 months despite conventional therapy, which includes maximal surgical resection, adjuvant temozolomide chemotherapy and radiotherapy (1). Immunotherapy using immune checkpoint inhibitors (ICI) has revolutionized cancer treatment but GBM patients have not yet benefited from this breakthrough (2). Adjuvant α-PD-1 therapy has not proven effective and neoadjuvant α-PD-1 therapy results in only a modest improvement in immune activation (3, 4). ICI therapy ‘releases the brakes’ on T cell activity, and therefore strictly relies on a preexisting T cell response towards the tumor. The lack of response in GBM is likely due to the scarcity of tumor antigen-directed T cells within the tumor microenvironment (TME).

Priming of naïve tumor antigen-directed T cells in lymphoid organs occurs when their cognate antigens are presented on the surface of professional antigen-presenting cells (APCs). While lymphatic vessels have been found within the meninges (5), there are no lymphatic vessels present within the brain parenchyma. Instead, fluid transport is mediated by the glymphatic system, a waste clearance system consisting of perivascular tunnels formed by astroglial cells, which does not allow for the migration of APCs (6). As such, the mechanisms that enable the priming of glioma antigen-directed T cells have not yet been elucidated, but likely rely on the transportation of antigens *via* the glymphatic system to the meningeal lymphatics. The antigens can then be taken up by meningeal APCs, which migrate through the dural lymphatics to cervical lymph nodes for priming of naïve T cells (5, 7, 8). Brain tumor immunity and the response to ICI can be improved through ectopic expression of vascular endothelial growth factor (VEGF)-C, which enhances lymphangiogenesis in the dura mater and thereby antigen transport to cervical lymph nodes in mouse models of glioma (9, 10). T cells primed against tumor-associated antigens then travel through the blood circulation and extravasate into the TME *via* activated tumor vasculature (11). This process, referred to as the cancer-immunity cycle, is less efficient in the central nervous system (CNS) than in the periphery.

The presence of tertiary lymphoid structures (TLS) in association with brain tumors (12, 13) suggests that these structures may serve as alternative sites for antigen presentation and T cell priming. Since the formation of TLS is a dynamic process that can be manipulated, this offers exciting new possibilities for enhancing the priming of brain tumor-reactive T cells. In this review, we briefly summarize the current understanding of the immunosuppressive GBM microenvironment, the formation and function of TLS in the CNS, and the putative implications for GBM immunity and immunotherapy.

## The Immune Microenvironment of GBM

The brain as an immune-privileged organ is a notion of the past: it is rather an actively regulated site of immune surveillance maintained by the meningeal lymphatic system (5, 14). Communication with the immune system differs significantly between the various CNS compartments, which are separated by specific brain barriers [reviewed in (15)]. Immune cells can readily enter the CNS through the subarachnoid space *via* the leptomeningeal vessels as well as the highly vascularized choroid plexus (16, 17). However, the immune trafficking in and out of the brain parenchyma is strictly controlled by the blood-brain barrier (BBB). The BBB consists of specialized brain microvascular endothelial cells and pericytes as well as the astrocyte endfeet and basal lamina, which comprise the glia limitans (15). This tight regulation is necessary to protect the brain from damaging inflammation, but it is also a significant hurdle for efficient immune responses against brain cancer.

Tumors can be classified as immune ‘desert’, ‘excluded’ or ‘inflamed’ based on the extent of infiltrating cytotoxic CD8^+^ T lymphocytes (CTLs) (18). Inflamed tumors are more likely to respond to ICI therapy, and accordingly a higher abundance of tumor-infiltrating CD8^+^ T cells is associated with improved prognosis and is a predictor of clinical outcome in GBM (19). However, the majority of GBM tumors are ‘immune-desert’ and essentially lack CTLs due to a number of tumor-related factors. Indeed, these tumors are poorly immunogenic as a result of low mutational burden and a scarcity of professional APCs in the GBM TME, which leads to decreased tumor antigen presentation (20). Furthermore, downregulation of MHC-I expression on the tumor cells limits their recognition by cytotoxic T cells (20). Newly diagnosed GBM patients may exhibit lymphopenia (21) due to the sequestration of T cells in the bone marrow (22). This results from GBM-induced downregulation of the G protein-coupled receptor S1P1, which controls egression of T cells from the secondary lymphoid organs (22). Low T cell levels are further exacerbated by conventional GBM treatments (21, 23).

The vasculature plays a critical role in immune cell extravasation into tissues. Infiltration of T cells across the BBB depends on the multistep process of lymphocyte diapedesis, mediating capture, rolling, adhesion and transendothelial migration, and subsequent re-activation of T cells by APCs in the perivascular space in order to cross the glia limitans [reviewed in (24)]. In GBM, the inflammatory milieu compromises the integrity of the BBB (25) but the vasculature is highly abnormal, which is in part due to pro-angiogenic factors such as VEGF in the TME (26, 27). This likely contributes to the poor T cell infiltration observed in GBM, as increased VEGF signaling in endothelial cells can reduce leukocyte recruitment by inhibiting the expression of adhesion molecules and chemokines required for T cell recruitment (28–30). Notably, vessel phenotype is heterogeneous in GBM and varies both between patients and within a single tumor (31). Single cell mRNA sequencing of endothelial cells isolated from human GBM demonstrated that while activated endothelial cells are detected in some patients, the majority of endothelial cells within the tumor are angiogenic and express low levels of adhesion molecules and chemokines (31).

In addition to their low abundance, T cells in GBM patients typically have a lower activation and proliferation status (32), are profoundly exhausted (33) and frequently express natural killer (NK) cell-related inhibitory receptors that further suppress their anti-tumor activity (23). Myeloid cells also contribute to T cell dysfunctionality in GBM. The immune-microenvironment of GBM is composed predominantly of highly plastic glioma-associated macrophages, which increase in number with tumor grade and are associated with poor prognosis (34, 35). Glioma-associated macrophages promote T cell anergy in GBM due to deficits in expression of costimulatory molecules and cytokines (36). Furthermore, professional APCs such as dendritic cells (DCs) are few in number in the CNS (37, 38), and their function can be impaired by tumor-derived factors in the GBM microenvironment. Fibrinogen-like protein 2 and other glioma-derived factors can impair differentiation of DCs in both the GBM microenvironment and tumor-draining lymph nodes by multiple mechanisms, including the overexpression of Nrf2 (39, 40). Furthermore, prostaglandin E2 produced by glioma cells can enhance interleukin (IL)-10 production in DCs, leading to increased immunosuppression (41). Each of these mechanisms ultimately leads to reduced effector T cell activation.

Overall, by establishing such a complex immunosuppressive ecosystem, GBMs can effectively evade immune recognition.

## Tertiary Lymphoid Structures in Association With Brain Tumors

TLS are ectopic aggregates of lymphoid and stromal cells that are transiently formed in non-lymphoid environments in association with chronic inflammatory conditions, including autoimmunity and cancer (42). The definition of a TLS is not conclusive and varies across studies, but they are generally accepted to be non-encapsulated aggregates of B cells and T cells. The maturity of TLS is believed to range from loose lymphoid clusters to highly organized structures resembling secondary lymphoid organs (SLOs), containing defined B cell follicles with active germinal centers as well as T cell zones (43). Other components of TLS can include DCs, follicular DCs (fDCs) and high endothelial venules (HEVs) which specialize in the recruitment of naïve lymphocytes (43). The mechanisms of TLS formation may vary in different biological systems. Similar to SLOs, the formation of TLS can be initiated by immune cells which take on the role of lymphoid tissue inducer cells (LTis), such as B cells and Th17 cells (44, 45). These cells secrete factors such as lymphotoxin-αβ and stimulate lymphotoxin-β-receptor (LTβR)-expressing cells that function as lymphoid tissue organizer cells (LTos). As a result, cytokines and chemokines are secreted that attract immune cells and induce angiogenesis (46). Once organized and mature, TLS encompass all cell types that are necessary for the priming of lymphocytes.

**Figure 1:**
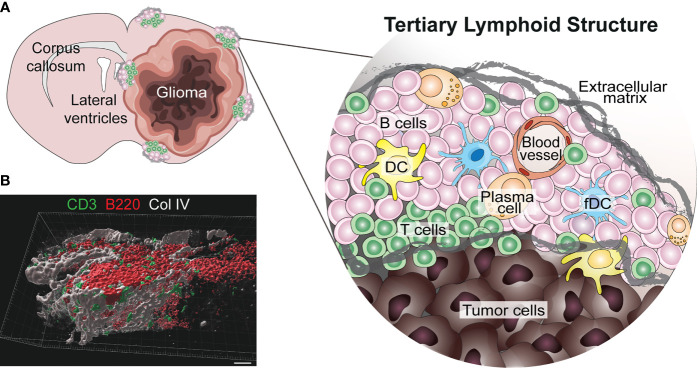
Known location and composition of TLS in murine glioma. **(A)** The image shows a schematic representation of the location and composition of TLS in murine glioma models (12). TLS form in the meningeal and ventricular regions of the tumor-bearing hemisphere, either in direct contact with the tumor or in its proximity. These structures form around blood vessels and are composed of B cells, T cells, dendritic cells (DCs), follicular DCs (fDCs) and a few plasma cells. Interestingly, murine glioma-associated TLS are surrounded by a network of extracellular matrix. **(B)** A 3D rendering of a cortical TLS (identified by B220^+^ B cells in red and CD3^+^ T cells in green) formed in a treatment-naïve GL261 glioma-bearing mouse, indicating how the structure is surrounded by a network of collagen IV (Col IV) in grey (scale bar 30 µm).

**Figure 2:**
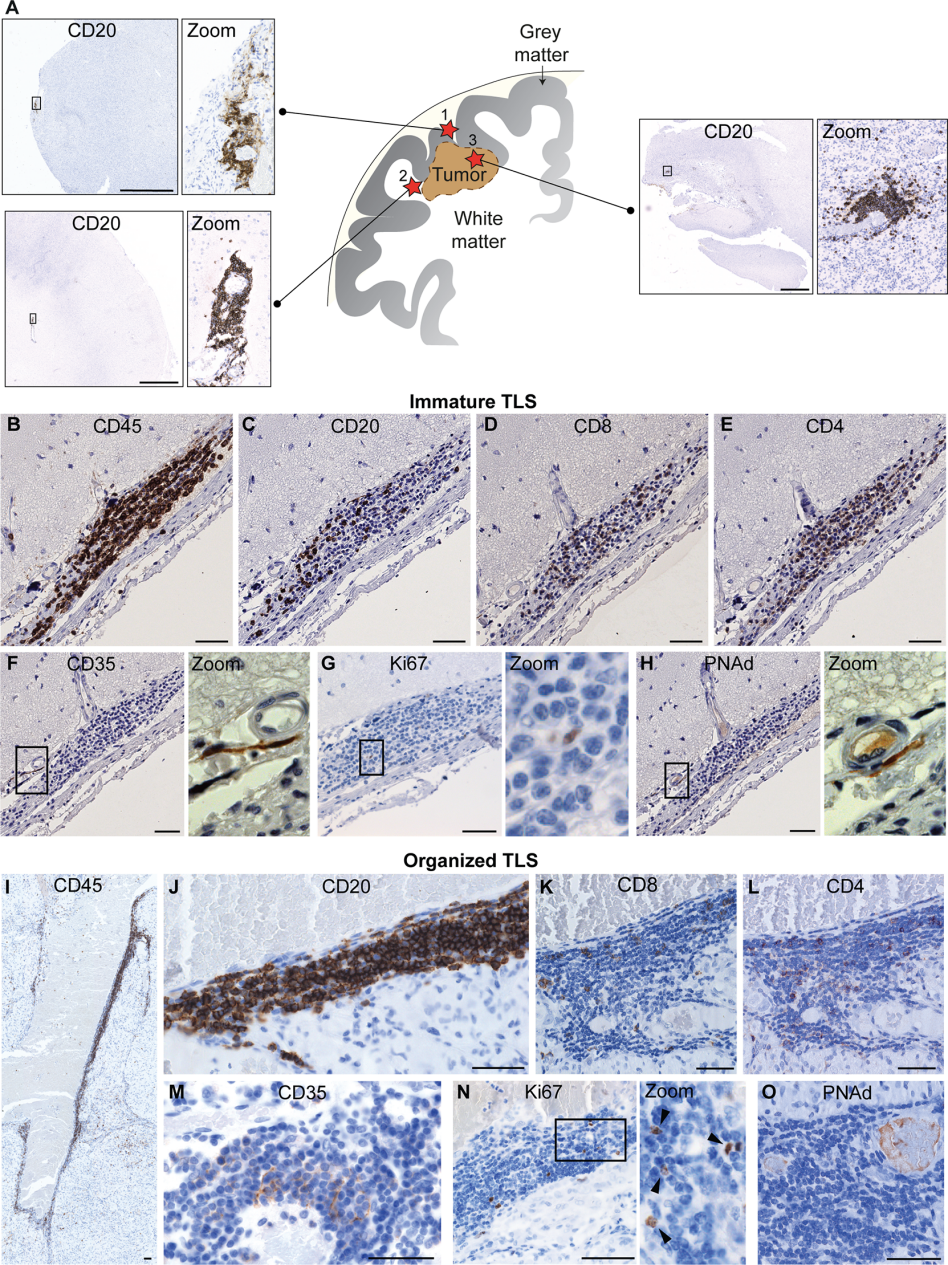
Known location and composition of TLS in human glioma. **(A)** The image shows a schematic representation of the known location of TLS in human glioma. To date, TLS have been identified in three main locations in glioma patients: (1) in direct proximity of the meningeal tissue, (2) in the white matter close to the tumor and (3) within the tumor tissue. A representative image of a TLS (identified by CD20^+^ B cell staining) is shown for each of the three locations. Scale bars: 2 mm. **(B–O)** illustrate the main cellular components of immature and organized TLS, respectively. While immature TLS contain dispersed B cells, organized TLS are characterized by a tight B cell core. Both immature and organized TLS contain CD8^+^ and CD4^+^ T cells and CD35^+^ follicular dendritic cells (fDCs), include proliferating cells, and form around PNAd^+^ high endothelial venules. Scale bars: 50µm. All images shown in this figure are reproduced from the original publication - *van Hooren, Vaccaro et al., Nature Communications, 2021* (12) under the terms of the Creative Commons Attributions License (CC BY).

TLS are present in many types of solid tumors and are generally associated with positive prognosis and improved response to immunotherapy (42, 47, 48). Furthermore, they have recently been observed in treatment-naïve human gliomas (12). TLS were also found in untreated glioma-bearing mice, and their formation was augmented by agonistic CD40 antibody therapy (αCD40) (12). Similarly, the delivery of an adenovirus expressing the CD40 ligand to murine brainstem tumors resulted in B cell aggregation in the surrounding meninges which was reminiscent of TLS (13). In orthotopic CT-2A and GL261 models, TLS were found close to the meninges or ventricles of the tumor-bearing hemisphere, but not always in direct contact with the tumor mass (12) (**Figure 1A**). Glioma-associated murine TLS contained B cells, T cells, DCs and fDCs, similar to those found in peripheral tumors, but were uniquely encapsulated by extracellular matrix molecules such as collagen and fibronectin, and exhibited an elongated morphology, likely due to the anatomical location (12) (**Figure 1**). TLS were also present in a subset of human WHO grade II-IV gliomas, not only in meningeal regions but also in the white matter proximal to the tumor as well as within the tumor tissue itself (12) (**Figure 2**). The TLS in human gliomas formed around peripheral node addressin (PNAd)-expressing vessels resembling HEVs (**Figure 2**). Interestingly, the presence of TLS in human gliomas correlated with higher infiltration of T cells (12), which may suggest local TLS-associated priming and expansion of tumor-infiltrating lymphocytes.

## TLS in CNS Autoimmunity – Similarities and Differences

TLS observed in autoimmune diseases of the CNS show some similarities and differences to those discovered in glioma, which may provide insight into their development and function in the brain cancer setting.

Multiple sclerosis (MS) is a neuroinflammatory autoimmune disease in which TLS have been described and are thought to exacerbate the characteristic autoreactive immune responses against CNS self-antigens. TLS containing B cell follicles, T cell zones and a network of fDCs have been observed in about 40% of secondary-progressive MS patients (49, 50). Interestingly, patient samples from earlier MS stages exhibited no TLS-like structures, emphasizing that chronic inflammatory signals are required for their development. Meningeal immune infiltrates containing both B cells and T cells were identified, but had no defined zones, follicles or fDC networks (45, 51). The presence of this infiltration correlated with increased levels of neurite loss and demyelination (45, 51), just as the presence of TLS in secondary-progressive MS patients was associated with underlying cortical damage and accelerated clinical disease (49, 50). This supports the popular hypothesis that TLS act as local sites for reinforcement of autoreactive immune responses in MS patients.

TLS were also observed in experimental autoimmune encephalomyelitis (EAE), the murine model of MS, where their formation was induced by Th17 cells (52). As with TLS in murine glioma, those in EAE were encapsulated by collagen fibers that extended into the structure, resembling the collagen-lined passages within lymph nodes (52). TLS in MS patients were observed exclusively in the meninges and were not found in the healthy or diseased parenchyma (49, 50), suggesting that proximity to the meningeal layer may be required for these structures to form. The MS TLS were closely associated with inflamed blood vessels which were not positive for the HEV marker PNAd (49, 53), while HEVs are largely unreported in EAE. An exception to this is a study on a B cell-dependent model of EAE in which both PNAd^+^ and MAdCAM-1^+^ HEVs were reported in TLS, however the majority of these structures formed in the cerebellum and the few within the cerebrum were confined to the ventricles (54). Therefore, it is of note that PNAd^+^ HEVs were present in cerebral human glioma TLS, which formed not only in the meninges but also in the white matter and tumor tissue (12) (**Figure 2**). This contrasts with the hypothesis that TLS can only form in the meninges and suggests that there may be multiple mechanisms for the formation and maintenance of TLS in the brain.

Ocular lymphoid clusters have been characterized in spontaneous murine and equine models of autoimmune uveitis, an autoimmune disease involving the attack of the healthy neuroretina (55–57). In the murine setting, distinct zones of B cells and T cells as well as the presence of fDC networks defined these clusters as TLS (55, 56). The structures were B cell-dominated (55, 56), but did not consistently associate with more severe disease as they do in MS (49, 50, 55). In earlier disease stages, the presence of organized TLS correlated with retained visual acuity (55). Interestingly, the TLS became more diffuse and disorganized as the disease progressed, and mice with these structures had poorer visual function than those without (55). This indicates that TLS may not uniformly function to progress autoimmune diseases, but that functional, well-organized TLS may hold autoimmune attacks at bay. In comparison, lymphocytic aggregates in the equine setting were T cell-rich and contained very few B cells, but were still defined by the authors as TLS (57). The relation between presence of these structures and disease severity was not determined, and the functions of different types of ectopic lymphoid clusters including TLS and other lymphoid aggregates should be further elucidated.

## The Potential Role of Stromal Niches in CNS TLS Formation

To date, it remains unclear which cells initiate and maintain TLS development in the inflamed CNS, and why they form in close proximity to meningeal tissues. The answers to these questions may be intertwined and connected to the unique organization of stromal cells in the CNS.

CNS stromal cells include fibroblasts, lymphatic endothelial cells (LECs), blood endothelial cells, pericytes and choroid epithelial cells, which are uniquely compartmentalized within specific stromal niches (58). Fibroblasts and LECs are selectively present within the meninges, indicating that they could be involved in the induction or maintenance of meningeal TLS. Both cell types have been implicated in TLS establishment during chronic inflammation in other organs (59–61). A recent study showed that dural LECs are involved in the regulation of brain tumor immunity (10), however their potential role in the formation of CNS TLS has not yet been investigated. The presence of TLS in a murine model of MS was associated with an increased proportion of meningeal PDPN^+^PDGFRα^+^PDGFRβ^+^ fibroblastic reticular cells, which expressed LTβR and CXCL13 (62), localized within meningeal TLS and were closely connected to a network of fibronectin and reticular fibers, similar to in lymph nodes. Likewise, it was demonstrated that these mice formed spinal cord TLS which were encapsulated by collagen (52). This data suggests that meningeal fibroblasts may produce lymphoid chemokines and extracellular matrix networks that support meningeal TLS formation during chronic inflammation.

CNS immune responses are generally initiated in the meninges, which are rich in immune cells, have access to the lymphatic system through the dura mater, and contain postcapillary venules that support immune cell trafficking (63). Vessels of the choroid plexus, which comprise the blood-CSF barrier, can also be readily activated upon systemic inflammation and participate in recruiting immune cells into the CNS (64). This may explain why CNS TLS are mostly found in the meninges or choroid plexus but not within the parenchyma, where immune cell recruitment is more strictly regulated by the BBB (12, 49, 50, 62). An exception to this is TLS in human glioma, which can also form in the cortical space close to the tumor around HEVs (12). Thus, it is possible that the formation of HEVs can allow TLS to form in locations other than the meninges. HEV formation has been associated with an active ongoing immune response, and can be enhanced by Treg depletion in peripheral tumors (65, 66). In murine glioma, HEV formation was induced by treatment with a vascular targeting peptide delivering LIGHT/TNFSF14 or when using a lymphotoxin β receptor agonist, and was further enhanced by anti-VEGF and ICI therapy (67, 68). HEV formation was associated with an accumulation of T cells, however it was not investigated whether these lymphoid aggregates included other TLS-related cell types, or if they were reminiscent of the antigen-presenting niches recently described to maintain stem-like T cells in tumors (69). The relative importance of HEV formation and different lymphoid niches for immune response in glioma is an important area of further investigation. Another possibility is that the cortical TLS have a direct connection to meningeal tissue through Virchow-Robin spaces, which are perivascular spaces lined by pia mater and fibroblastic cells that originate in the leptomeninges and penetrate the cortex surrounding venules or arterioles (70–72).

Altogether, current experimental evidence indicates that multiple CNS stromal cell types could be involved in the formation of meningeal and cortical TLS. Elucidating their specific functions may offer new targets for regulation of TLS induction in CNS pathologies.

## Future Directions – Can TLS Induction in the CNS Improve Anti-Tumor Immunity?

The correlation of intratumoral TLS formation with positive prognosis and patient survival in many forms of cancer (42) has led to attempts to induce TLS as a form of immunotherapy (73, 74). TLS are associated with enhanced T cell presence in human tumors, and similar observations in GBM patients indicate that TLS induction may be beneficial in this setting. Notably, enhanced TLS formation was observed in glioma-bearing mice treated with αCD40, confirming that induction of TLS is feasible in brain cancer (12). However, αCD40 also induced T cell hypofunction. This was associated with a systemic upregulation of regulatory B cells, which was not related to TLS induction. Therefore, the potential benefit of TLS induction in GBM should be investigated using other inducers. Moreover, it is possible that not only the presence but also the composition of TLS is important for guiding the immune response. Indeed, the existence of regulatory T cells within TLS has been associated with suppression of anti-tumor immunity (75). A deeper knowledge of how TLS composition affects anti-GBM immune responses is necessary to enable the development of therapies that can efficiently induce TLS and consequently boost T cell priming and activation.

Strong immune activation within the CNS is associated with certain risks, including oedema and autoimmunity. CNS oedema is limited by the cranium and can have devastating effects: the swelling can lead to raised intracranial pressure, impaired function and even death. CNS tumors such as GBM display increased vascular permeability, giving rise to peritumoral oedema (76) which would likely be enhanced by strengthening immune activation. Additionally, immunotherapy aiming for TLS induction in the meningeal space may lead to local activation of autoreactive lymphocytes and thus the attack of normal CNS tissue. Such adverse events could resemble MS, where formation of TLS has been associated with subpial cortical damage and disease progression (77). Similarly, TLS formation is often observed in affected organs in other autoimmune diseases and has been associated with both autoantibody production and disease progression (78), but this has not been studied in glioma. Corticosteroids can be used to reduce symptoms of oedema, inflammation and autoimmune attack in the CNS, but can also dampen the effects of immunotherapy and TLS formation. For GBM patients who received ICI therapy, the use of dexamethasone was associated with shorter survival (79). Furthermore, corticosteroid treatment during chemotherapy negatively affected the development of TLS and abrogated their prognostic value in lung cancer patients (43). Therefore, treatment with corticosteroids should be used with caution in association with immunotherapy as this may counteract TLS formation and result in reduced anti-tumor immune responses.

In conclusion, GBM-associated TLS correlate with an influx of T cells in the tumors, indicating that adaptive immune responses can form locally in the CNS in this setting. However, many questions remain to be answered. Is TLS formation in GBM associated with a survival benefit? Which are the molecular cues for TLS formation in GBM? How does this connect with immune activation and T cell infiltration in the tumors? The answers to these questions can enable the development of new strategies to enhance immune responses in brain cancer.

## Author Contributions

All authors listed have made a substantial, direct, and intellectual contribution to the work and approved it for publication.

## Funding

This work was supported by grants from the Swedish Cancer Society [20 1008 PjF], [20 1010 UsF], [190184Pj]; the Swedish Childhood Cancer Fund [PR2018-0148], [PR2020-0167]; the Swedish Research Council [2020-02563], [2019-01326]; Knut and Alice Wallenberg foundation [KAW 2019.0088]. MR was supported by a postdoctoral grant from the Swedish Childhood Cancer Fund [TJ 2019-0014]. AD was supported by a Senior Investigator Award from the Swedish Cancer Society [CAN 2015/1216].

## Conflict of Interest

The authors declare that the research was conducted in the absence of any commercial or financial relationships that could be construed as a potential conflict of interest.

## Publisher’s Note

All claims expressed in this article are solely those of the authors and do not necessarily represent those of their affiliated organizations, or those of the publisher, the editors and the reviewers. Any product that may be evaluated in this article, or claim that may be made by its manufacturer, is not guaranteed or endorsed by the publisher.
